# Highly Efficient Esterification of Ferulic Acid Under Microwave Irradiation

**DOI:** 10.3390/molecules14062118

**Published:** 2009-06-10

**Authors:** Nian-Guang Li, Zhi-Hao Shi, Yu-Ping Tang, Bao-Quan Li, Jin-Ao Duan

**Affiliations:** 1 Jiangsu Key Laboratory for TCM Formulae Research, Nanjing University of Chinese Medicine, Nanjing, Jiangsu 210046, China; E-mails: linianguang@163.com (N-G.L.); libaoquan121@163.com (B-Q.L.); 2 Division of Organic Chemistry, China Pharmaceutical University, Nanjing, Jiangsu 211198, China; E-mail: sszh163@163.com (Z-H.S.); 3 Department of Medicinal Chemistry, Nanjing University of Chinese Medicine, Nanjing, Jiangsu 210046, China

**Keywords:** esterification, ferulic acid, microwave irradiation, highly efficient

## Abstract

A highly efficient synthesis of alkyl ferulates under microwave irradiation is described. The time of these reactions ranged from 3 to 5 minutes, which was much shorter than the traditional synthetic methods, and the alkyl ferulates were obtained in higher yields.

## 1. Introduction

It is well established that under normal conditions, there is a steady state balance between pro-oxidants and antioxidants, which is necessary to ensure the optimal efficiency of antioxidant defenses. However, when the rate of free radical generation exceeds the capacity of antioxidant defenses, oxidative stress ensues, with severe damage to the cell [1]. Therefore, the use of radical scavengers should be of critical importance to prevent and cure many diseases such as ischemia-reperfusion injury, neurodegenerative disorders and surgical organ transplantation [2]. Recent evidence has demonstrated that ferulic acid is a scavenger of hydroxyl and peroxyl radicals in both brain cells and macrophages and easily forms a resonance-stabilized phenoxy radical [3,4,5]. However, ferulic acid has only limited solubility in hydrophobic media, which reduces its antioxidant effects in inhibiting auto-oxidation of fats and oils [6]. The strategy of esterification of hydrophilic ferulic acid with lipophilic molecules, such as aliphatic alcohols, could be employed to alter its solubility in a hydrophobic medium. Indeed, it has been found that the hydrophobic alkyl ferulate derivatives have a higher antioxidative activity than ferulic acid for the prevention of oxidation of linoleic acid in a bulk system [7].

In the classical method of esterification ferulic acid was refluxed with alcohols in the presence of various catalysts, such as concentrated sulfuric acid, hydrogen chloride, boron trifluoride, aluminum chloride, trifluoroacetic anhydride, polyphosphate ester, neodymium oxide, dicyclohexylcarbodiimide, graphite bisulfate, etc [8,9,10], but it was difficult to avoid the disadvantages of using these catalysts, such as long reaction times, low yield, expensive reagents and tedious operation. For instance, in 2007, Rault’s group [11] described a classical esterification of ferulic acid in the presence of methanol and sulfuric acid (95%) to afford methyl ferulate, and the same procedure was used in the presence of ethanol to afford ethyl ferulate, however, the reaction required 24 h in this procedure.

Ferulic acid is heat-sensitive and susceptible to oxidation, which has made enzymatic syntheses of its esters more attractive than chemical syntheses. However, enzymatic syntheses also have many disadvantages including low yields, time consumption, solvent requirements, etc [12,13]. For example, in 2006, Yoshida and co-workers [14] continuously synthesized 1-pentyl, 1-hexyl and 1-heptyl ferulates at 60-90 ºC, however, the reactor system they used, in which a column packed with ferulic acid powder and another column packed with immobilized *Candida antarctica* lipase particles were connected in series, was very special and the reaction must continue for at least 40 h in order to obtain the alkyl ferulates in high yield.

In recent years, microwave-assisted reactions have received a great deal of attention, because reactions under microwave irradiation are in general not only faster than with conventional heating methods, but also potentially more efficient, clean, and safe [15,16]. Further improvements have also been reported whereby microwave-assisted reactions can offer enhanced reaction rates, higher yields, and greater selectivity for the targeted product under milder reaction conditions [17]. In 2002, Lee’s group reported a facile and efficient method for the conversion of ferulic acid to the corresponding alkyl carboxylates with trialkyl orthoacetate under microwave irradiation under solvent-free and neutral reaction conditions [18], but their method could only be applied to trimethyl orthoacetate and triethyl orthoacetate because trialkyl orthoacetates with long lined carbon chains or branched carbon chains could not be easily synthesized. Because alcohols are a type of polar solvent, they can absorb microwaves very efficiently [17], so we have therefore attempted the use of alcohols as alkylation reagents for the esterification of ferulic acid under the microwave irradiation. The aim of this study was to establish an efficient strategy of synthesizing alkyl ferulates under the microwave irradiation, and to evaluate its efficiency in comparison with the traditional method.

## 2. Results and Discussion

### 2.1. Optimization of the catalyst for the synthesis of ethyl ferulate

**Scheme 1:**
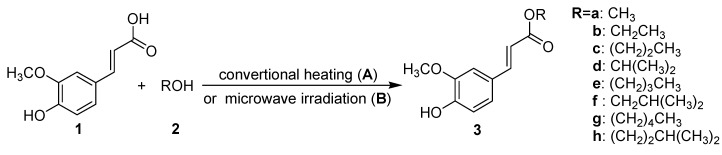
Syntheses of alkyl ferulates

In our research, we selected ethanol (**2b**) as a representative alcohol to optimize the reaction conditions (cf. Scheme 1A. First, we tried this esterification without any catalysts, and ferulic acid (**1**) was reacted with the ethanol (**2b**) directly under microwave irradiation with the power set at 200 W (Table 1, Runs 1-2). Unfortunately, there was no indication of product formation even when the reaction time was extended for 10 mins. To make this reaction proceed easily, we added conc. sulfuric acid to the reaction mixture as catalyst. This time the esterification in the presence of 10 mol% H_2_SO_4_ in ethanol was found to be complete after 5 mins at 88 ºC, giving the corresponding ethyl ferulate (**3b**) in 94% yield (Table 1, Run 7). When the amount of H_2_SO_4_ was increased to 12 mol%, the yield of the product was reduced to 86% (Table 1, Runs 8); this could be because the ethyl ferulate was hydrolyzed if the amount of H_2_SO_4_ used was excessive. However, when the amount of H_2_SO_4_ was decreased (2, 4, 6, 8 mol%), the reaction proceeded more slowly, and the yields of ethyl ferulate (**3b**) were reduced to 56, 70, 86 and 90%, respectively, even when the reaction was carried out for 5 mins at 88 ºC (Table 1, Runs 3-6).

**Table 1:** Optimization of the catalyst concentration for the synthesis of ethyl ferulate (**3b**)^a^.

Run	Cat.(mol %)	Time^c ^(min)	Yield^d^ (%)
1	0	5	0
2	0	10	0
3	2^b^	5	56
4	4^b^	5	70
5	6^b^	5	86
6	8^b^	5	90
7	10^b^	5	94
8	12^b^	5	86

^a ^Reaction conditions: Ferulic acid (**1**, 1 mmol), ethanol (**2b**, 5 mL), temperature 88 °C; ^b ^Conc. sulfuric acid as the catalyst; ^c ^Monitored by TLC; ^d ^Isolated yield, purity confirmed by MS and ^1^H- NMR.

### 2.2. Optimization of the temperature for the synthesis of ethyl ferulate

Reaction temperature played a crucial role in this microwave-assisted esterification. We found that the increase of the temperature remarkably accelerated the reaction (Table 2, Runs 1-5). A high yield was obtained within 5 mins when the esterification was carried out in ethanol at 88 °C (20°C higher than the boiling point of ethanol) (Table 2, Run 5). The esterification at 48 °C for 5 mins gave only a 40% yield of desired product (Table 2, Run 1). However, for this esterification, a higher temperature was unfavorable as the desired ethyl ferulate (**3b**) was hydrolyzed (Table 2, Runs 6-7).

**Table 2:** Optimization of the temperature for the synthesis of ethyl ferulate (**3b**)^a^.

Run	Temp. (ºC)	Time^b ^(min)	Yield^c^ (%)
1	48	5	40
2	58	5	54
3	68	5	76
4	78	5	86
5	88	5	94
6	98	5	90
7	108	5	83

^a ^Reaction conditions: Ferulic acid (**1**, 1 mmol), ethanol (**2b**, 5 mL), Conc. sulfuric acid (10 mol%); ^b ^Monitored by TLC; ^c ^Isolated yield, purity confirmed by MS and ^1^H-NMR..

### 2.3. Optimization of the reaction time for the synthesis of ethyl ferulate

The effect of the reaction time was also examined (Table 3, Runs 1-6). From the results we could see that as the reaction time was extended from 2 to 7 mins, the yield of the desired product ethyl ferulate (**3b**) improved from 56% to 94% (Table 3, Runs 1-4). However, when the reaction time exceeded 4 mins, the yield was not affected so much (Table 3, Runs 5-6).

**Table 3:** Optimization of the reaction time for the synthesis of ethyl ferulate (**3b**)^a^.

Run	Time^b ^(min)	Yield^c^ (%)
1	2	56
2	3	94
3	4	94
4	5	93
5	6	94
6	7	94

^a ^Reaction conditions: Ferulic acid (**1**, 1 mmol), ethanol (**2b**, 5 mL), Conc. sulfuric acid (10 mol%), Temperature (88 ºC); ^b ^Monitored by TLC; ^c ^Isolated yield, purity confirmed by MS and ^1^H-NMR.

### 2.4. Optimization of the molar ratio of ferulic acid to ethanol for the synthesis of ethyl ferulate

Finally, we investigated the effect of the molar ratio of ferulic acid to ethanol in this esterification (Table 4, Runs 1-8). It was found that when the molar ratio was more than 1:6, the targeted product of ethyl ferulate was obtained in almost quantitative yield (Table 4, Runs 6-8). Decreasing the molar ratio to 1:1, the ethyl ferulate was obtained in only 30% after this esterification was continued for 3 mins (Table 4, Run 1), and as the molar ratio of ferulic acid to ethanol increased, the yield of ethyl ferulate was also enhanced (Table 4, Runs 2-6).

**Table 4:** Optimization of the molar ratio of ferulic acid to ethanol for the synthesis of ethyl ferulate (**3b**)^a^.

Run	Molar Ratio (1 : 2b)	Time^b ^(min)	Yield^c^ (%)
1	1 : 1	3	30
2	1 : 2	3	54
3	1 : 3	3	83
4	1 : 4	3	88
5	1 : 5	3	92
6	1 : 6	3	94
7	1 : 7	3	94
8	1 : 8	3	93

^a ^Reaction conditions: Ferulic acid (**1**, 1 mmol), Conc. sulfuric acid (10 mol%), Temperature (88ºC); ^b ^Monitored by TLC; ^c ^Isolated yield, purity confirmed by MS and ^1^H-NMR.

### 2.5. Synthesis of alkyl ferulates under conventional heating and microwave irradiation

After we have optimized the catalyst, temperature, reaction time and the molar ratio of ferulic acid to alcohol for this esterification, we applied this methodology to the reactions between ferulic acid and other alcohols, and compared the efficiency of microwave-assistance with conventional heating (Table 5). The result showed that the reaction times for conventional heating were always very long, the longest reaction time being 28 h, as in the case of **3h** (Table 5, Run 8). The reaction time became much longer as the number of atoms in the alcohol increased, such as in the case of **3b** (Table 5, Run 2) and **3c** (Table 5, Run 3), where the reaction times were 8 h and 14 h, respectively. For alcohols with same atom numbers, the reaction time for alcohols with branched carbon chains was much longer than those with linear carbon chains, for instance, the reaction time of **3c** was 14 h (Table 5, Run 3), while the reaction time of **3d** was 20 h (Table 5, Run 4). Another interesting phenomenon was that similarly the yields of the alkyl ferulates turned to be much lower as the atom numbers of the alcohol became more, such as in the case of **3e** (73%) (Table 5, Run 5) and **3a** (79%) (Table 5, Run 1), and the yield of the alkyl ferulates with branched alkyl chains was much lower than those with linear alkyl chains, which could be observed from the case of **3g** (58%) (Table 5, Run 7) and **3h** (46%) (Table 5, Run 8). These differences in the reaction time and yields between reactions with branched and linear alcohols may be due to steric hindrance in the alcohols with branched carbon chains [19]. When microwave irradiation was applied to these esterifications of ferulic acid, all the reaction times decreased to several minutes, even for the alcohols with long carbon chains or branched chains, such as in the case of **3g** (Table 5, Run 7) and **3h **(Table 5, Run 8), where the reaction time for synthesis of these two ferulates was only 5 min under the microwave irradiation, especially for **3h**, the reaction time was reduced from 28 h to 5 min, while the yield increased from 46% to 91%, and furthermore, all the yields achieved in the synthesis of ferulates under microwave irradiation were above 90% (Table 5). This interesting phenomenon may be attributed to an increased equilibrium constant under microwave irradiation, as in the reaction vessel, the reactive pressure must be very high under microwave irradiation when the vessel is sealed, so the equilibrium constant might be increased because this equilibrium constant is in directly proportional to pressure [20].

**Table 5:** Synthesis of alkyl ferulates under conventional heating and microwave irradiation.

Run	Sub.	Prod.	Conventional heating^a^			Microwave irradiation^b^		
			Temp.	Time^c^	Yield^d^	Temp.	Time^c^	Yield^d^
1	**2a**	**3a**	reflux	8 h	79	75 ºC	3 min	95
2	**2b**	**3b**	reflux	8 h	81	88 ºC	3 min	94
3	**2c**	**3c**	reflux	14 h	77	107 ºC	4 min	94
4	**2d**	**3d**	reflux	20 h	69	92 ºC	4 min	93
5	**2e**	**3e**	reflux	18 h	73	128 ºC	4 min	93
6	**2f**	**3f**	reflux	24 h	63	118 ºC	4 min	92
7	**2g**	**3g**	reflux	22 h	58	148 ºC	5 min	93
8	**2h**	**3h**	reflux	28 h	46	142 ºC	5 min	91

^a ^Reaction conditions: **1 **(1 mmol), alcohol (5 mL), Conc. sulfuric acid (10 mol%); ^b ^Reaction conditions: **1 **(1 mmol), alcohol (6 mmol), Conc. sulfuric acid (10 mol%); ^c ^Monitored by TLC; ^d^Isolated yield, purity confirmed by MS and ^1^H-NMR.

## 3. Experimental

### 3.1. General

All reagents were commercially available and used directly. The ^1^H-NMR spectra were recorded on a Bruker AV 300 spectrometer using CDCl_3_ as the solvent and TMS as the internal standard. Chemical shifts are reported in parts per million (ppm). The ESI-MS were obtained on Agilent 1946A-MSD. IR spectra were recorded on a Nicolet Impact 410 instrument. Elemental analysis was performed on an Elementar Vario EL III analyzer.

### 3.2. General procedure for the esterification of ferulic acid with conventional heating

To a stirred mixture of ferulic acid (**1**, 970 mg, 5 mmol) in alcohol (5 mL) was added concentrated sulfuric acid (0.027 mL, 0.5 mmol), and the reaction mixture was refluxed until the ferulic acid had completely reacted, as indicated by TLC. After cooling to 25°C, ethyl acetate was added and the mixture washed with water and brine. The ethyl acetate layer was dried over MgSO_4_, filtered and concentrated under reduced pressure. The residue was purified by silica gel column chromatography eluted with petroleum ether-EtOAc (8:1, v/v) to afford the corresponding ferulates.

### 3.3. General procedures for the esterification of ferulic acid with microwave irradiation

Concentrated sulfuric acid (0.027ml, 0.5 mmol) was added dropwise to a stirred mixture of ferulic acid (970 mg, 5 mmol) in alcohol (30 mmol) and the reaction mixture was refluxed in a sealed Discover (CEM, USA) reaction vessel under microwave irradiation; the power was set at 200W, and the temperature was set at some 20°C above the boiling point of the alcohol, and the pressure was set at 180 psi.

### 3.4. Analytical and Spectroscopic Data for Some Representative Compounds and New Compounds

*(E)-Methyl 3-(4-hydroxy-3-methoxyphenyl)acrylate (**3a**)* [11]: White solid. ^1^H-NMR δ 3.80 (s, 3H), 3.91 (s, 3H), 6.29 (d, *J* = 15.9 Hz, 1H), 6.88 (d, *J* = 8.0 Hz, 1H), 7.04 (m, 2H), 7.62 (d, *J* = 15.9 Hz, 1H); ESI-MS *m/z*: 209 [M +H]^+^ (100); IR (KBr) 3383, 2950, 2844, 2645, 2356, 1599, 1169, 816, 567 cm^-1^; Anal. Calcd for C_11_H_12_O_4_: C, 63.45; H, 5.81; Found: C, 63.41; H, 5.85. 

*(E)-Ethyl 3-(4-hydroxy-3-methoxyphenyl)acrylate (**3b**)* [11]: White solid. ^1^H-NMR δ 1.31 (t, 3H), 3.92 (s, 3H), 4.25 (q, 2H), 6.29 (d, *J* = 15.9 Hz, 1H), 6.91 (d, *J* = 8.0 Hz, 1H), 7.06 (m, 2H), 7.61 (d, *J* = 15.9 Hz, 1H); ESI-MS *m/z*:223 [M +H]^+^ (100); IR (KBr) 3401, 2979, 2357, 1699, 1599, 1518, 1456, 1378, 1169, 1033, 816, 569 cm^-1^; Anal. Calcd for C_12_H_14_O_4_: C, 64.85; H, 6.35; Found: C, 64.82; H, 6.39. 

*(E)-Propyl 3-(4-hydroxy-3-methoxyphenyl)acrylate (**3c**)* [19]: White solid. ^1^H-NMR δ 1.01 (t, 3H), 1.75 (m, 2H), 3.94 (s, 3H), 4.15 (q, 2H), 6.31 (d, *J* = 15.9 Hz, 1H), 6.93 (d, *J* = 8.0 Hz, 1H), 7.05 (m, 2H), 7.63 (d, *J* = 15.9 Hz, 1H); ESI-MS *m/z*: 237 [M +H]^+^ (100); IR (KBr) 3366, 2965, 2638, 2355, 1698, 1519, 1261, 816, 568 cm^-1^; Anal. Calcd for C_13_H_16_O_4_: C, 66.09; H, 6.83; Found: C, 66.01; H, 6.88. 

*(E)-Isopropyl 3-(4-hydroxy-3-methoxyphenyl)acrylate (**3d**)* [19]: Light brown solid. ^1^H-NMR δ 1.32 (d, *J* = 6.2Hz, 6H), 3.92 (s, 3H), 5.14 (m, 1H), 6.27 (d, *J* = 15.9 Hz, 1H), 6.91 (d, *J* = 8.0 Hz, 1H), 7.03 (m, 2H), 7.60 (d, *J* = 15.9 Hz, 1H); ESI-MS *m/z*: 237 [M +H]^+^ (100); IR (KBr) 3383, 2978, 2359, 1692, 1597, 1514, 1462, 1426, 1375, 1275, 1180, 902, 810, 656, 566 cm^-1^; Anal. Calcd for C_13_H_16_O_4_: C, 66.09; H, 6.83; Found: C, 66.02; H, 6.89. 

*(E)-Butyl 3-(4-hydroxy-3-methoxyphenyl)acrylate (**3e**)* [19]: Light brown oil. ^1^H-NMR δ 0.97 (t, 3H), 1.45 (m, 2H), 1.69 (m, 2H), 3.93 (s, 3H), 4.20 (t, 2H), 6.29 (d, *J* = 15.9 Hz, 1H), 6.92 (d, *J* = 8.0 Hz, 1H), 7.04 (m, 2H), 7.61 (d, *J* = 15.9 Hz, 1H); ESI-MS *m/z*: 251 [M +H]^+^ (100); IR (KBr) 3559, 2849, 2331, 2066, 1955, 1859, 1063 cm^-1^; Anal. Calcd for C_14_H_18_O_4_: C, 67.18; H, 7.25; Found: C, 67.11; H, 7.20. 

*(E)-Isobutyl 3-(4-hydroxy-3-methoxyphenyl)acrylate (**3f**)* [19]: Light brown oil. ^1^H-NMR δ 0.98 (d, *J* = 6.8Hz, 6H), 2.03 (m, 1H), 3.92 (s, 3H), 3.98 (d, *J* = 6.6 Hz, 2H), 6.30 (d, *J* = 15.9 Hz, 1H), 6.91 (d, *J* = 8.0 Hz, 1H), 7.05 (m, 2H), 7.62 (d, *J* = 15.9 Hz, 1H); ESI-MS *m/z*: 251 [M +H]^+^ (100); IR (KBr) 3440, 2975, 2657, 2353, 2069, 1953, 1859, 1634, 1443, 1223, 1130, 982, 819, 571 cm^-1^; Anal. Calcd for C_14_H_18_O_4_: C, 67.18; H, 7.25; Found: C, 67.13; H, 7.28. 

*(E)-Pentyl 3-(4-hydroxy-3-methoxyphenyl)acrylate (**3g**)* [19]: Light brown oil. ^1^H-NMR δ 0.88 (t, 3H), 1.36 (m, 4H), 1.70 (m, 2H), 3.91 (s, 3H), 4.20 (t, 2H), 6.28 (d, *J* = 15.9 Hz, 1H), 6.90 (d, *J* = 8.0 Hz, 1H), 7.04 (m, 2H), 7.60 (d, *J* = 15.9 Hz, 1H); ESI-MS *m/z*: 265 [M +H]^+^ (100); IR (KBr) 3422, 2335, 2065, 1860, 1636, 1447, 1049, 819, 571 cm^-1^; Anal. Calcd for C_15_H_20_O_4_: C, 68.16; H, 7.63; Found: C, 68.11; H, 7.68. 

*(E)-Isopentyl 3-(4-hydroxy-3-methoxyphenyl)acrylate (**3h**)* [19]: Yellow oil. ^1^H-NMR δ 0.95 (d, *J* = 6.6 Hz, 6H), 1.56 (m, 2H), 1.81 (m, 1H), 3.91 (s, 3H), 4.22 (t, 2H), 6.29 (d, *J* = 15.9 Hz, 1H), 6.91 (d, *J* = 8.0 Hz, 1H), 7.07 (m, 2H), 7.61 (d, *J* = 15.9 Hz, 1H); ESI-MS *m/z*: 265 [M +H]^+^ (100); IR (KBr) 3373, 2958, 2356, 1699, 1519, 1163, 816, 569 cm^-1^; Anal. Calcd for C_15_H_20_O_4_: C, 68.16; H, 7.63; Found: C, 68.19; H, 7.66.

## 4. Conclusions

In summary, an efficient microwave-assisted esterification of ferulic acid with alcohols was developed for the first time. The reported procedure affords high yields in shorter reaction times, and the results of the present study should be of value to synthesize other esters.
